# Our Proverbs

**Published:** 1877-09

**Authors:** 


OUR PROVERBS.—Listen if you would learn. Be silent if you would be safe. Inquire about your neighbor before you travel. The first of wisdom is the fear of God. The world is carrion, and its followers dogs. Poverty without debt is independence. Long experience makes large wit. The sluggard becomes a stranger to God, and an acquaintance with Indigence. By six qualities may a fool be known : Anger without cause, speed without profit, change without motive, inquiry without an object, putting trust in a stranger, and wanting capacity to discriminate between a friend and a foe.

